# A Conserved Tryptophan in the Envelope Cytoplasmic Tail Regulates HIV-1 Assembly and Spread

**DOI:** 10.3390/v14010129

**Published:** 2022-01-12

**Authors:** Xenia Snetkov, Tafhima Haider, Dejan Mesner, Nicholas Groves, Schuyler B. van Engelenburg, Clare Jolly

**Affiliations:** 1 Division of Infection and Immunity, University College London, London WC1E 6BT, UK; x.snetkov@alumni.ucl.ac.uk (X.S.); tafhima.haider.15@alumni.ucl.ac.uk (T.H.); dejan.mesner.15@ucl.ac.uk (D.M.); 2 Molecular and Cellular Biophysics Program, Department of Biological Sciences, University of Denver, Denver, CO 80210, USA; Nicholas.Groves@du.edu (N.G.); Schuyler.VanEngelenburg@du.edu (S.B.v.E.)

**Keywords:** HIV-1, Env, viral assembly, cell–cell, T cell

## Abstract

The HIV-1 envelope (Env) is an essential determinant of viral infectivity, tropism and spread between T cells. Lentiviral Env contain an unusually long 150 amino acid cytoplasmic tail (EnvCT), but the function of the EnvCT and many conserved domains within it remain largely uncharacterised. Here, we identified a highly conserved tryptophan motif at position 757 (W757) in the LLP-2 alpha helix of the EnvCT as a key determinant for HIV-1 replication and spread between T cells. Alanine substitution at this position potently inhibited HIV-1 cell–cell spread (the dominant mode of HIV-1 dissemination) by preventing recruitment of Env and Gag to sites of cell–cell contact, inhibiting virological synapse (VS) formation and spreading infection. Single-molecule tracking and super-resolution imaging showed that mutation of W757 dysregulates Env diffusion in the plasma membrane and increases Env mobility. Further analysis of Env function revealed that W757 is also required for Env fusion and infectivity, which together with reduced VS formation, result in a potent defect in viral spread. Notably, W757 lies within a region of the EnvCT recently shown to act as a supporting baseplate for Env. Our data support a model in which W757 plays a key role in regulating Env biology, modulating its temporal and spatial recruitment to virus assembly sites and regulating the inherent fusogenicity of the Env ectodomain, thereby supporting efficient HIV-1 replication and spread.

## 1. Introduction

Virus assembly is a well-orchestrated event in which all components must be temporally and spatially recruited to the right place at the right time to regulate successfully infectious viral egress and spreading infection. For the lentivirus human immunodeficiency virus type-1 (HIV-1), replication predominantly takes place in CD4^+^ T cells, with virus assembly and budding occurring at the plasma membrane (PM) [1]. HIV-1 has two major structural proteins: the envelope glycoprotein (Env) that engages CD4 and co-receptor (CCR5 or CXCR4) and mediates fusion of the viral lipid membrane and the target cell plasma membrane to mediate entry, as well as the Gag p55 polyprotein that undergoes cleavage by the viral protease during budding, a step that is required for viral maturation and infectivity, producing the Gag components capsid, matrix, nucleocapsid, and p6. Env and Gag are trafficked independently to sites of lentiviral assembly at the plasma membrane. During HIV-1 assembly, trimers of Gag matrix (MA) assemble at the PM, forming a lattice of hexamers that directly interact with phosphatidylinositol-4,5-bisphosphate (PIP2) in the membrane [2,3], with Gag capsid (CA) comprising the viral core. Recent super-resolution imaging has shown that the Gag lattice forms early during the course of viral assembly, with Env arriving at a later stage [4]. Self-multimerisation of Gag, lattice-associated Env, and the host cell ESCRT machinery together appear to drive virus assembly and budding [5,6,7,8]. Whether the EnvCT interacts with Gag MA, how exactly Env gets incorporated into virions and whether Env plays any role in regulating virus assembly remain unclear [1,9].

Env is composed of the gp120 domain that mediates receptor binding, the gp41 domain that is composed of an extracellular and transmembrane domain, and a long cytoplasmic tail of 150 amino acids. Conservation of the long lentiviral envelope cytoplasmic tail (EnvCT) suggests the presence of key determinants within the CT that are essential for efficient lentiviral replication and spread [10]. By contrast to lentiviruses, most related retroviruses have an EnvCT that is considerably shorter at 30–40 amino acids [11]. The importance of the EnvCT for lentiviral replication is best demonstrated by the observation that when the C-terminal EnvCT is truncated (CT∆144 virus), HIV-1 is unable to spread in T cell lines and primary CD4^+^ T cells due to an Env incorporation defect [10]. Notably, the first structural data of the EnvCT revealed that the majority of the long HIV-1 EnvCT resides buried in the membrane [12]. The N terminus of the EnvCT consists of a short, membrane-proximal, unstructured loop containing a YxxL endocytic motif that binds the clathrin adaptor, AP-2 [13,14]. Rapid endocytosis of Env from the plasma membrane via the YxxL and an additional C-terminal dileucine motif limits the amount of Env present of the surface of infected cells [13,14,15,16,17]. This is thought to protect infected cells from detection by the immune system and limit the amount of Env packaged into nascent virions to aid immune evasion [18,19]. Following endocytosis from the plasma membrane, retrograde and recycling trafficking pathways are proposed to coordinate Env recycling back to the cell surface [15,17,20,21,22]. Previous work has shown that endocytosis mediated by Y712 helps direct virus budding to the basolateral membrane in epithelial cells and also influences the site of virus budding in T cells, presumably by allowing endocytosed Env to access intracellular trafficking machinery for correct sorting back to virus assembly sites [20,23,24]. Thus, while the biology of the EnvCT remains to be fully elucidated, the absolute dependence on the EnvCT for infectious virus assembly in T cells argues against a passive incorporation model and highlights the importance of the EnvCT in HIV-1 replication and spread. Thus, it is likely that the HIV-1 EnvCT contains additional undefined motifs that regulate Env trafficking, localisation to virus assembly sites and virion incorporation.

HIV-1 spreads between CD4^+^ T cells via two distinct mechanisms: either by cell-free infection or by direct cell-to-cell transmission (reviewed in [25]). The latter is the dominant mode of HIV-1 spread in vitro [26,27,28,29], whereas in vivo the tight packing of CD4^+^ T cells in lymphoid organs and their propensity for frequent interactions provide opportunity for HIV-1 to exploit cell–cell spread to rapidly disseminate from one cell to another [30]. Cell–cell spread occurs following physical contact between HIV-1-infected donor T cells and the uninfected target T cells and is termed the virological synapse (VS) [27]. VS formation is characterised by the recruitment of HIV-1 Env and Gag to sites of cell–cell contact, leading to polarised virus assembly and budding that is focused towards the target cell [27,28,31,32]. Thus, cell–cell spread is an active and regulated process that is triggered by cell–cell contact, but the mechanisms by which Env and Gag are directed to the VS and how virus assembly is orchestrated during cell–cell spread remain to be fully-elucidated. Understanding this is important, and it provides a tractable and valuable experimental model to interrogate how HIV-1 assembly is temporally and spatially regulated and reveal novel viral determinants.

Here, we have sought to provide molecular insight into the processes of HIV-1 assembly and spread, specifically the role of the EnvCT. To this end, we have identified a highly conserved tryptophan residue at position 757 within the first helix of the EnvCT (LLP2) that mediates HIV-1 spread between T cells. Specifically, we show that W757 regulates Env and Gag recruitment to virus assembly sites at the VS, limits lateral diffusion of Env within the plasma membrane and regulates Env fusogenicity. Thus, our data identify a highly conserved site within the EnvCT that plays a key role in regulating multiple Env functions.

## 2. Materials and Methods

### 2.1. Cells and Viral Constructs

Jurkat CE6.1 T cells were grown in RPMI 1640 medium (Thermo Fisher Scientific, Waltham, MA, USA) supplemented with 10% fetal calf serum (FCS, Labtech, Heathfield, UK) and 100 U/mL penicillin–streptomycin (Thermo Fisher Scientific, Waltham, MA, USA). HEK 293T and HeLa TZM-bl cell lines were grown in DMEM medium (Thermo Fisher Scientific, Waltham, MA, USA) supplemented with 10% FCS and 100 U/mL penicillin–streptomycin. Peripheral blood mononuclear cells (PBMC) were isolated from buffy coats from healthy donors using Ficoll Paque Plus (Sigma, St. Louis, MI, USA) according to manufacturer’s instructions. CD4^+^ T cells were isolated by negative selection using MojoSort Human CD4 T Cell Isolation kit (Biolegend, San Diego, CA, USA). Primary CD4^+^ T cells were maintained in complete RPMI 1640 medium supplemented with 10 IU/mL IL-2 (Centre for AIDS Reagents (CFAR), National Institute of Biological Standards and Controls, UK). Primary CD4^+^ T cells were activated for 4–5 days in T25 flasks coated with 5 µg anti-CD3 antibody (clone OKT3, Biolegend, San Diego, CA, USA) in the presence of 2 µg/mL soluble anti-CD28 antibody (clone CD28.2, Biolegend, San Diego, CA, USA). The CEM-A cell line was obtained through NIH AIDS Reagent Program, Division of AIDS, NIAID, NIH: CEM-A from Dr. Mark Wainberg and Dr. James McMahon, CEM-CL10 [33]. Complete growth media for the CEM-A cell line was prepared by combining 10% fetal bovine serum (Corning, NY, USA), 2 mM L-glutamine (Corning, NY, USA), 1% hypoxanthine thymidine (HT) solution (Corning, NY, USA), and penicillin–streptomycin (Corning, NY, USA) into Roswell Park Memorial Institute (RPMI) medium (Corning, NY, USA). HIV-1 NL4.3 (donated by Dr. Malcolm Martin) was obtained from the CFAR. Site-directed mutagenesis was carried out on full-length replication-competent pNL4.3 by 15 amplification cycles using Pfu polymerase with the following primers: EnvCT W757A (GTGAACGGATCCTTAGCACTTATCGCGGACGATCTGCGGAGCCTGTG); W757Y (GTGAACGGATCCTTAGCACTTATCTACGACGATCTGCGGAGCCTGTG); W757F (GTGAACGGATCCTTAGCACTTATCTTCGACGATCTGCGGAGCCTGTG), followed by Dpn1 (NEB) digest and sequence verification. The NL4.3 EnvCT mutant (∆CT) is described in [34]. Viral stocks were generated by co-transfecting 293T cells with NL4.3 plasmid and VSV-G envelope plasmid using Fugene 6 (Promega, Madison, WI, USA). After 48 h, virus was harvested and infectivity assayed by serial titration on HeLa TZM-bl reporter cells using Bright-Glo (Promega, Madison, WI, USA).

### 2.2. Antibodies

Antibodies used for flow cytometry: anti-HIV-1 p24-PE (KC57-RD1 Beckman Coulter), anti-HIV-1 Env clone PGT151 (gift from Laura McCoy, UCL) and 2G12 (Polymun, Klosterneuburg, Austria); Secondary antibody: anti-Human IgG Cy5 (polyclonal Bethyl). Antibodies for western blotting: anti-HIV-1 gp120 rabbit antisera (donated by Dr S. Ranjibar and obtained from the CFAR), anti-HIV-1 gp41 mAb 246-D (donated by Dr S. Zoller-Pazner and Dr M. Gorny, obtained from the CFAR) and anti-HIV-1 Gag rabbit antisera (donated by DR G. Reid and obtained from the CFAR). Secondary antibodies: anti-Rabbit IgG (ab216773, Abcam), anti-Mouse IgG (ab216775, Abcam, Cambridge, UK) and anti-Human IgG (926-32232, Licor, Lincoln, NE, USA). The BG18-QD625 antibody fab fragment probe was produced recombinantly for single-particle tracking as previously described [35].

### 2.3. HIV-1 Infections and Cell–Cell Spread Assays

T cells were infected with 50,000 TCID50/million cells with VSV-G pseudotyped HIV-1 by gravity infection for 4 h. For cell–cell spread assays, HIV-1-infected T cells (donors) were analysed for Gag expression by flow cytometry 48 h post-infection (p.i.). Uninfected T cells (targets) were labelled with 2.5 μM Cell Proliferation Dye eFluor 450 (Thermo Fisher Scientific, Waltham, MA, USA) according to manufacturer’s instructions. HIV-1-infected Gag+ donors and dye-labelled targets were mixed in 1:1 ratio, incubated at 37 °C for indicated time points and analysed by flow cytometry using a BD LSR Fortessa X-20 (BD Biosciences Systems & Reagents Inc., San Jose, CA, USA) or Calibur cytometer (BD Biosciences Systems & Reagents Inc., San Jose, CA, USA). Data were analysed using FlowJo software (BD Biosciences Systems & Reagents Inc., San Jose, CA, USA).

### 2.4. Virus Release and Infectivity Assays

HIV-1-infected T cells were incubated for up to 24 h, and virus-culture supernatants were harvested. Total virus released into the supernatant was measured by qPCR to quantify the supernatant reverse transcriptase (RT) activity using SG-PERT assay [36], and RT activity was normalised to the number of Gag+ (HIV-1-infected) cells. Virion infectivity was determined by luciferase assay using HeLa TZM-bl reporter cells. To determine particle infectivity, RLU infectivity values were normalised to RT activity.

### 2.5. Viral Fusion Assay

NL4.3 WT or W757A was co-transfected with BlaM-Vpr (Addgene, Watertown, MA, USA) and pAdVAntage (Promega, Madison, WI, USA) into 293T cells to generate virions packaging BlaM-Vpr. Virus was concentrated by ultracentrifugation and quantified by SG-PERT. Next, 10^11^ pU RT of virus was used to infect target 10^6^ Jurkat cells for 4 h by gravity infection, and the BlaM-Vpr assay was performed as described [37].

### 2.6. Immunofluorescence

Uninfected Jurkat cells (target cells) were labelled with 10 μM CellTracker Blue CMAC dye (Thermo Fisher Scientific, Waltham, MA, USA) according to the manufacturer’s instructions. Infected Jurkat cells (donor cells) were mixed with target cells in a 1:1 ratio and incubated with (nonblocking) anti-Env antibody for 1 h at 37 °C on poly-l-lysine-coated coverslips [27]. Cells were fixed with 4% paraformaldehyde, permeabilised with 0.1% Triton X-100 (Sigma, St. Louis, MI, USA) and stained with anti-Gag serum. Primary antibodies were detected with appropriate fluorescent secondary antibodies. Coverslips were mounted with ProLong Gold Antifade mounting solution (Thermo Fisher Scientific, Waltham, MA, USA). Images were acquired on a DeltaVision Elite image restoration microscope (Applied Precision, Washington, DC, USA) with softWoRx 5.0 software (Applied Precision, Washington, DC, USA). Envelope enrichment at the VS was measured by the increase in fluorescence intensity at the contact site over intensity at a distal membrane region, normalised to background intensity. Image processing and analysis were performed using FIJI software [38].

### 2.7. Simultaneous Super-Resolution and Single-Particle Tracking of HIV-1 Biogenesis

All imaging, processing and analysis was done per the optimised methodology of Groves et. al., 2020 [35]. Briefly, CEM-A T cells in complete RPMI medium (10% fetal bovine serum; 2 mM L-glutamine; 1% penicillin/streptomycin, and 1% hypoxanthine/thymidine; Corning, NY, USA) were seeded on 25 mm No. 1.0 glass coverslips (Warner Instruments; Hamden, CT, USA) and infected with VSV-G pseudotyped virus packaged with the pSV-NL4-3 HIV-1 reference genome, encoding the previously described CA-Skylan-S nanobody probe, with deletions in the gag-p6 late-domain, pol, vif, vpr and nef genes [35]. Forty-eight hours post-infection, cells were stained with anti-Env BG18-QD625 probe and coverslips were transferred to specimen holders and mounted on a custom-built ring-TIRF microscope with a live-cell chamber maintaining 37 °C and 5% CO_2_. Skylan-S and QD625 were simultaneously excited with a 473 nm laser, and fluorescence was detected by separation of wavelengths using a dichroic beamsplitter housed in a W-View Gemini image splitter (Hamamatsu, Hamamatsu City, Japan). As previously described, images were focused onto two halves of a liquid-cooled ORCA Fusion scientific-CMOS camera (C14440-20UP) (Hamamatsu, Hamamatsu City, Japan) streaming at 100 Hz [35]. Raw images were corrected for non-uniform pixel offset and split based on wavelength. Single-molecule localisations for both channels were fit using custom software (IDL, Harris Geospatial; Broomfield, CO, USA), and lateral chromatic aberration between channels was corrected using a high-density fiducial map collected after data acquisitions (100 nm TetraSpeck; Thermo Fisher Scientific, Waltham, MA, USA). Single-molecule localisations were passed to an automated package written in MATLAB (Natick, MA, USA). This software determined Gag assembly centroids and linked single-molecule Env trajectories from frame to frame. Tracks were split into ‘proximal’ and ‘distal’ classifications based on the proximity of their localisations to Gag bud centroids as previously described [35]; Linking parameters: 50 ms maximum time gap, 10 localisations per track minimum and 1 pixel (108.33 nm) maximum frame-to-frame distance between successive track positions The apparent trajectory diffusion coefficients (*D_apparent_*) and the slope of the moment scaling spectrum (*S_MSS_*) were computed by fit to a linear regression and weighted by localisation uncertainties. Statistical significance between log-transformed *D_apparent_* or *S_MSS_* parameters for Env genotypes was assessed using nonparametric *t*-tests and the statistical software package Prism. Nonparametric tests were selected as diffusion coefficient, and *S_MSS_* histograms are primarily lognormally distributed (also see supplemental data for complete histograms).

### 2.8. Envelope Recycling Assay

Infected Jurkat cells were stained with envelope-specific antibodies PGT151 Ab (5 μg/mL) and 2G12 (5 μg/mL) for 60 min at 37 °C in FCS-supplemented RPMI to allow for Env internalisation [39]. Cells were thoroughly washed in ice-cold FACS wash buffer, and surface Env was detected with anti-human-PE secondary antibody for 30 min on ice to measure Env surface levels at T_0min._ Cells were again washed extensively in ice-cold buffer and incubated with anti-human-Cy5 secondary antibody for 0 to 180 min at 37 °C in FCS-supplemented RPMI to label Env that is recycling back to the cell surface. Cells were fixed in 4% paraformaldehyde and analysed by flow cytometry.

### 2.9. Flow Cytometry

T cells were washed and incubated with antibodies (described above) at 4 °C for surface staining and cell viability using dye Zombie UV (Biolegend, San Diego, CA, USA). Primary antibodies were detected with anti-human IgG secondary antibody, fixed and analysed. To stain for intracellular antigens, cells were fixed with 4% paraformaldehyde and permeabilised with CytoPerm buffer (Biolegend, San Diego, CA, USA). Cells were analysed on BD LSR Fortessa X-20 cytometer (BD Biosciences Systems & Reagents Inc., San Jose, CA, USA). Compensation was performed using CompBeads (BD Biosciences Systems & Reagents Inc., San Jose, CA, USA) and calculated by FACSDiva software (BD Biosciences Systems & Reagents Inc., San Jose, CA, USA). Data were analysed using FlowJo software (BD Biosciences Systems & Reagents Inc., San Jose, CA, USA).

### 2.10. Western Blotting

Fifty micrograms of cell lysate and an equivalent volume of purified virus (purified by ultracentrifugation over sucrose) were separated by SDS-PAGE and analysed by western blotting using the following primary antibodies: anti-HIV-1 gp120, anti-HIV-1 gp41, anti-HIV-1 Gag, and anti-tubulin (described above). Primary antibodies were detected with appropriate fluorescent secondary antibodies and imaged with Odyssey Infrared Imager (Licor, Lincoln, NE, USA). Immunoblots were analysed with Image Studio Lite software (Licor, Lincoln, NE, USA).

### 2.11. Neutralisation Assays

Antibody and inhibitor neutralisation assay was performed as described previously [40]. Briefly, virus supernatants were incubated with serial dilutions of soluble CD4 (CFAR NIBSC), AMD3100 (CFAR, NIBSC), PGT151 [41] (gift from Laura McCoy, UCL) or T-20 fusion inhibitor (CFAR, NIBSC) and incubated at 37 °C for 1 h. The mixture was added to TZM-bl cells, and luciferase activity measured 48 h later using Bright-Glo substrate (Promega, Madison, WI, USA). Neutralisation was calculated as percent decrease in luciferase activity compared to virus-only control. IC_50_ values were calculated by nonlinear regression analysis (sigmoid curve interpolation) using Prism software (GraphPad, San Diego, CA, USA).

### 2.12. Statistical Analysis

Statistical significance was calculated where appropriate using a paired/unpaired student’s *t*-test or one-way ANOVA. Significance was assumed when *p* < 0.05. All statistical analyses were calculated using Prism 9 (GraphPad Prism, San Diego, CA, USA).

## 3. Results

### 3.1. A Conserved Tryptophan Residue in the EnvCT Is Required for HIV-1 Cell–Cell Spread and Virological Synapse Formation

**Figure 1:**
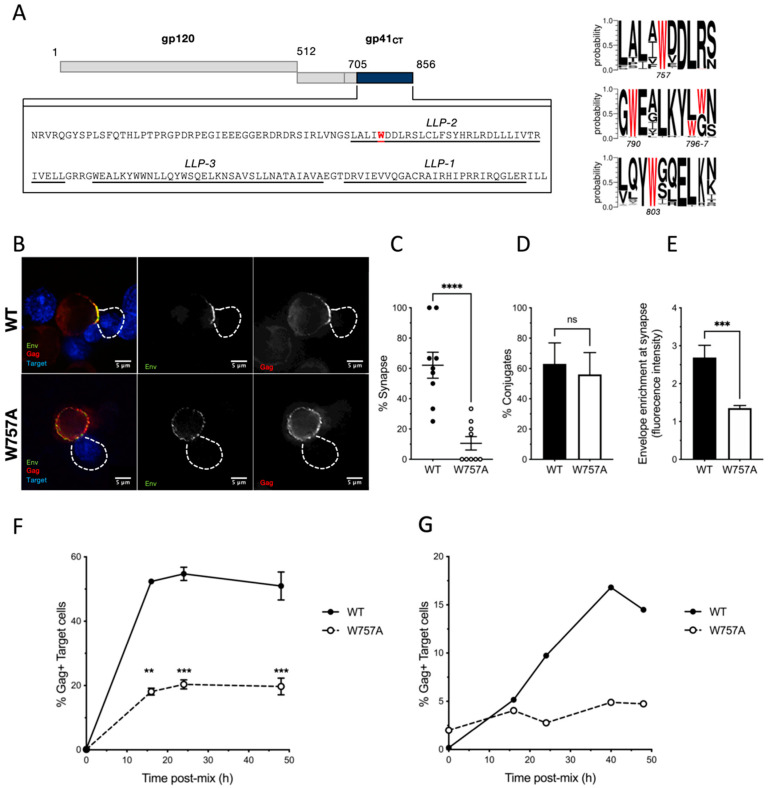
EnvCT W757A does not polarise to viral synapses and leads to a defect in cell–cell spread. (**A**) Schematic showing the HIV-1 NL4.3 EnvCT sequence and location of W757 in the LLP2 alpha helix. Logo plots (generated using WebLogo3) indicate the probability of 5916 HIV-1 Env sequences analysed in the LANL database containing a W residue at the indicated positions. (**B**) Virological synapse formation between Jurkat cells infected with WT NL4.3 (top) or the W757A mutant (bottom) and uninfected target cells. NL4-3 virus pseudotyped with VSVg to normalise viral entry was used to infect donor Jurkat cells. Representative image is an *xy* slice through the middle of a cell–cell contact between an infected donor cell (Env, green; Gag, red) and a target cell stained with a cell trace dye (blue). (**C**) Percent of contacts that exhibited virological synapse formation (defined by polarisation of both Env and Gag to the contact site). (**D**) Quantification of the percentage of infected donor cells (Gag+) in contact with target cells (blue). Data show the mean and SEM from *n* = 54–69 infected donor cells compared using a two-tailed paired *t*-test (ns, not significant; *** *p* < 0.001, **** *p* < 0.0001). € Signal intensity enrichment of Env at the synapse, relative to a synapse distal site, was determined from 20 images using FIJI software. (**F**,**G**) Infected donor cells were mixed in a 1:1 ratio with target cells (stained with cell trace far red), and viral spread was measured by intracellular Gag staining in target cells by flow cytometry. Data show the percent of Gag+ target cells over time. Jurkat-to-Jurkat spreading infection (**F**), and primary CD4^+^ T cell-to-autologous primary CD4^+^ T cell spreading infection (**G**). Data show the mean and SEM from four independent Jurkat cells experiments analysed using paired *t*-tests (ns, not significant; ** *p* <0.01, *** *p* < 0.001). For primary CD4^+^ T cells, a representative donor from three unique donors is shown.

**Figure 2:**
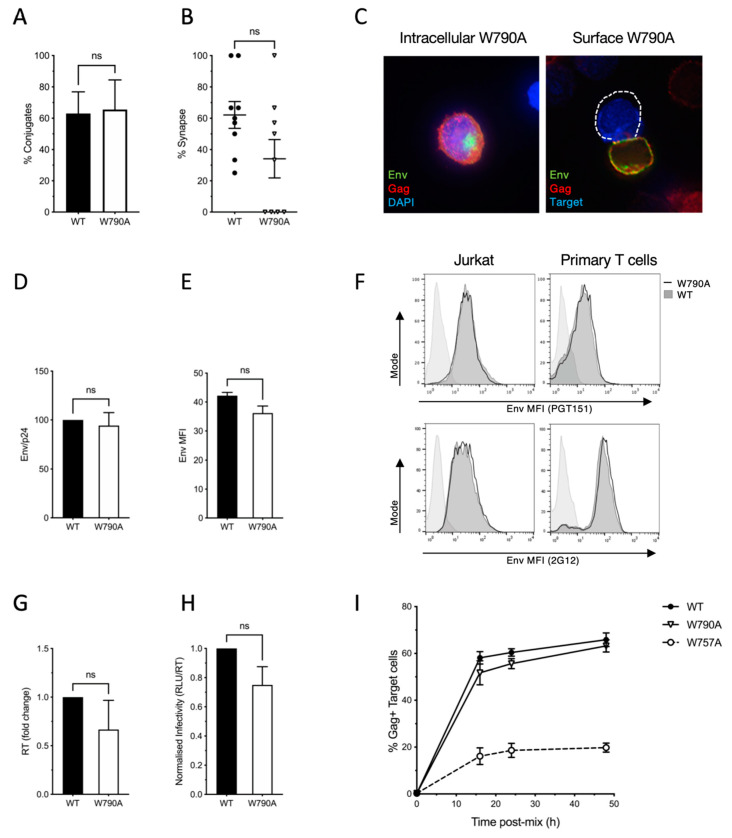
EnvCT W790A virus does not perturb HIV-1 Env function and viral cell–cell spread. (**A**) Alanine substitution of the tryptophan at HIV-1 envelope position 790 does not affect the ability of infected Jurkat cells to form conjugates with uninfected target cells. (**B**,**C**) EnvCT W790A-infected cells also form synapses to the same extent as WT HIV-1 do and result in no defect in both intracellular and surface localization of Env. (**D**) Quantification of Env (gp120) incorporation into virions produced in Jurkat cells, normalised to Gag (p24) density. (**E**) Quantification of surface Env MFI in infected Jurkat cells measured by flow cytometry analysis of cells stained with bNab PGT151. (**F**) Cell surface W790A Env staining (solid black line, unfilled) and WT virus (dark grey, filled) measured by flow cytometry staining using bNab PGT151 (top) or 2G12 (bottom). Uninfected control is shown as a light grey-filled histogram. (**G**,**H**) Budding of W790A virions from infected cells (**G**), and the relative infectivity (**H**) of W790A virus is equivalent to that of WT. (**I**) Spreading infection of W790A virus between Jurkat T cells over time (measured by flow cytometry analysis of the percentage Gag+ cells) is equivalent to that of WT virus, but is defective for W757A virus.

To identify novel determinants in EnvCT that play a role in HIV-1 replication and spread, we focused on tryptophan (W) residues that possess a large, bulky hydrophobic side chain and act as key mediators of protein–protein and protein–lipid interactions, including within the W-rich HIV-1 MPER [42,43]. The 150 amino acid HIV-1 EnvCT contains five tryptophan residues at positions 757, 790, 796, 797 and 803 (Figure 1A). Analysis of 5916 HIV-1 Env sequences from the Los Alamos database (http://www.hiv.lanl.gov/ accessed on 24 September 2020) revealed that three of these (W757, W790 and W803) are highly conserved amongst HIV-1 isolates (Figure 1A) [44,45]. We focused on the two tryptophan residues at W757 and W790, given that disruption of Y802-W803 has been previously characterised and shown to reduce Env incorporation and infectivity [46,47]. Using a mutagenesis approach, the first tryptophan at the beginning of the LLP-2 helix of the cytoplasmic tail of HIV-1 envelope (W757) was substituted for alanine in full-length replication-competent HIV-1 NL4.3 and the effects on viral replication and cell–cell spread were examined. To normalise infection (and obviate any differences in the proportion of HIV-1-infected cells that may bias analysis), HIV-1 wild type (WT) and the tryptophan mutant (W757A) were pseudotyped with VSV-G to achieve equivalent initial infection of T cells. We focused on cell–cell spread, which is the dominant mode of HIV-1 dissemination between T cells and takes place at the VS. HIV-1 infected Jurkat cells (donors) were infected with WT or W757A virus for up to 48 h, then mixed with dye-labelled uninfected Jurkat cells (targets), and cell–cell interactions and VS formation were quantified. Consistent with previous reports, co-culturing HIV-1 WT-infected donors and uninfected targets resulted in 62% of cell–cell contacts between an infected T cell and an uninfected T cell forming a VS, evidenced by polarisation of Env and Gag on infected cells to the sites of contact with a target cell (Figure 1B,C). Strikingly, substituting the tryptophan at position 757 with an alanine (W757A virus) abolished VS formation, resulting in loss of both Env and Gag recruitment to the contact zone (Figure 1B,C). Importantly, the frequency of cell–cell contacts formed between donor and target T cells remained unchanged, implicating a specific defect in VS formation and not an inability of cells to interact (Figure 1D) and form Env-CD4 dependent cell–cell adhesions. We did not re-validate the requirement for Env-CD4 in cell–cell contact as this is well established in previous work [27,28,29]. By contrast to WT Env, W757A Env displayed a more punctate, uniform distribution around the plasma membrane that was not enriched at the contact zone (Figure 1B,E). As expected, the inability of the HIV-1 Env W757A mutant to form VS resulted in a significant defect in HIV-1 cell–cell spread. Co-culture of WT-infected Jurkat T cells and uninfected target T cells resulted in 55% of target T cells becoming infected after 24 h, by contrast to W757A virus that was significantly less able to spread (Figure 1F and Supplementary Figure S1). Similar results were obtained when spreading infection was performed using primary CD4^+^ T cells; however, in this case the defect in W757A virus was even more pronounced, with no increase in viral spread to target cells evident up to 50 h post-infection (Figure 1G and Supplementary Figure S1). By contrast to the W757A mutant, the W790A mutant displayed no significant defect in VS formation or cell–cell spread (Figure 2). Therefore, we focused on understanding the specific requirement for W757 in Env biology and viral spread.

### 3.2. Single-Molecule Tracking of Env at Virus Assembly Sites

**Figure 3:**
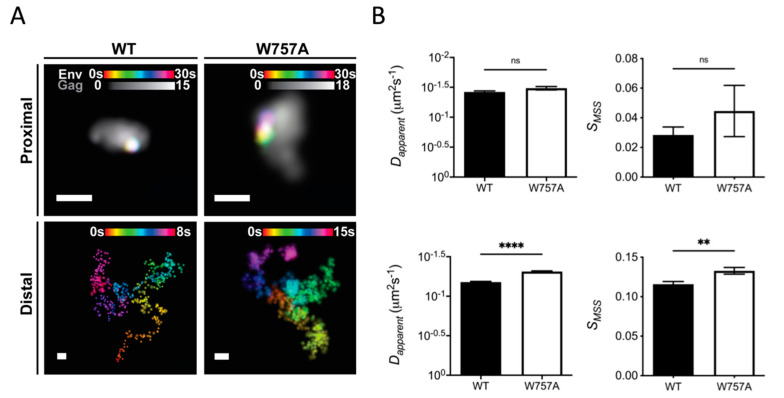
W757A EnvCT does not display a Gag lattice trapping defect at virus assembly sites. CEM-A adherent T cells were transduced with WT and W757A mutant envelope virus and imaged after 48 h. (**A**) Super-resolution reconstructions of Gag and lattice-proximal/-distal single-molecule localisations of Env are demonstrated (Scale bars = 200 nm). Arrows highlight highly confined single-molecule localisations of Env. Proximal and distal single-molecule localisations show the confined and freely diffusing nature of single Env trimers, respectively. (**B**) Wild-type and mutant Env trimers do not differ in mobility when lattice-proximal (top panels) (WT: $D_{apparent}=0.038{\;\mu m}^{2}\;s^{-1}$, $S_{MSS}=0.01$; W757A: $D_{apparent}=$ 0.033 $\mu^{2}\;s^{-1}$, $S_{MSS}\;$ = 0.045), whereas lattice-distal diffusion differs significantly between viruses (bottom panels) (WT: $D_{apparent}=0.066{\;\mu }^{2}\;s^{-1}$, $S_{MSS}=0.116$; W757A: $D_{apparent}=0.049{\;\mu }^{2}\;s^{-1},$ $S_{MSS}=0.133$). Statistical significance was determined by unpaired two-sample *t*-test ** *p* < 0.01, **** *p* < 0.0001 (proximal: $P_{D}=9.22\times 10^{-24}$, $P_{S_{MSS}}=0.29$; distal: $P_{D}=0.19$, $P_{S_{MSS}}=0.0016$) N = distal: N_WT_ = 3652, N_W757A_ = 2698).

To explain the defect in spreading infection of W757A virus, and the inability of Env to polarise to virus assembly sites at the VS, single-particle tracking was performed on Env trimers proximal and distal to super-resolved sites of virus assembly to interrogate the mobility of wild-type EnvCT and W757A mutants [35]. This approach has previously elucidated the dynamics of Env mobility at the cell surface, revealing that during nascent virus assembly, Env becomes trapped or corralled in the Gag lattice to facilitate incorporation into virions [4,48]. Importantly, the lateral diffusion of Env within and outside virus assembly sites was shown to be dependent on the EnvCT, as well as Gag MA [48]. Thus, we hypothesised that failure of Env W757A to localise to virus assembly sites and polarise to the VS may be explained by a lattice trapping defect, leading to inefficient incorporation of Env into the budding Gag lattice. Exploring this notion, single-molecule tracking of Env and super-resolution imaging of Gag assembly sites revealed that trimers of Env W757A were significantly more mobile in the PM compared to WT Env, with Env W757A diffusing more freely when distal to the Gag lattice (Figure 3A,B lower panels, and Supplementary Figure S2; *D_apparent_* = 0.07 ± 0.17 for Env WT versus *D_apparent_* = 0.05 ± 0.10 for Env W757, *p* < 0.0001; *S_MSS_* = 0.12 ± 0.21 for Env WT versus *S_MSS_* = 0.13 ± 0.22 for Env W757, *p* = 0.0017). By contrast, proximal W757A mutant trimers exhibited similar diffusion characteristics as lattice proximal WT Env trimers did (Figure 3A,B upper panels and Supplementary Figure S2; *D_apparent_* = 0.038 ± 0.06 for Env WT *D_apparent_* = 0.033 ± 0.03 for Env W757, *p* = 0.0693; *S_MSS_* = 0.03 ± 0.13 for Env WT versus *S_MSS_* = 0.05 ± 0.16 for Env W757, *p* = 0.3743), suggesting that W757A mutant trimers are not defective for lattice trapping and confinement. Taken together, these data suggest a role for W757 in the EnvCT in modulating Env movement within the PM.

### 3.3. Mutating W757 Impacts HIV-1 Particle Infectivity

**Figure 4:**
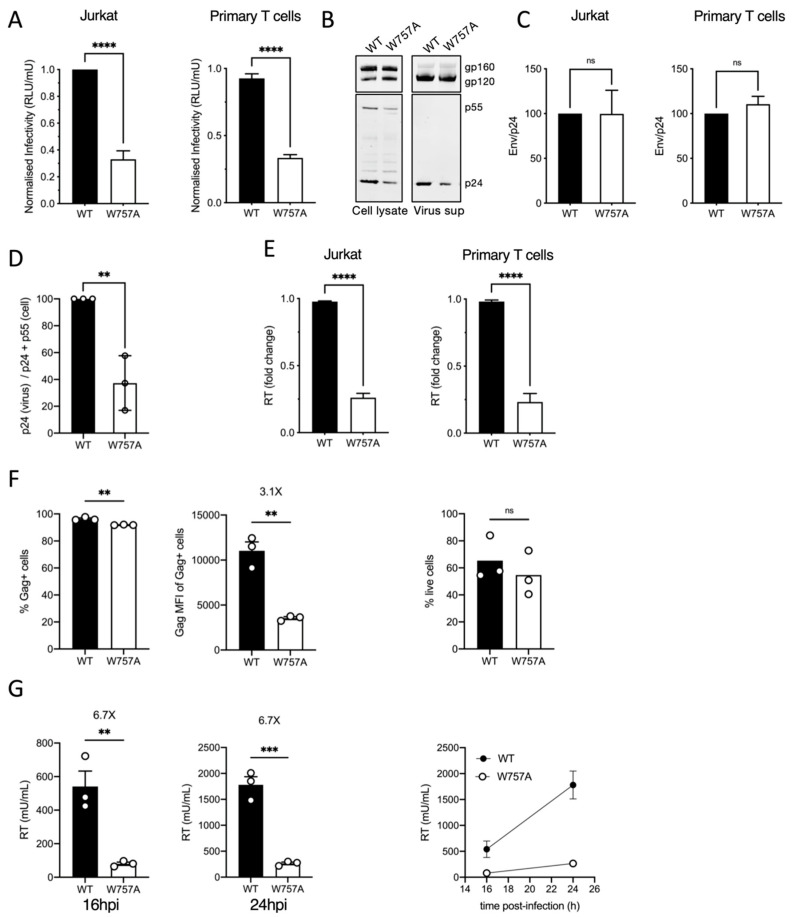
EnvCT W757 is required for HIV-1 infectivity and budding. (**A**) Infectivity of virus particles released 48 hpi from Jurkat and primary CD4^+^ T cells was measured by titrating virus on HeLa TZM-bl reporter cells and RLU normalised per RT unit to calculate relative particle infectivity. (**B**) Western blot of cell lysate and purified virus from infected primary CD4^+^ T cells at 48 hpi probed with anti-Env (gp120/gp160) and anti-Gag (p55/p24). (**C**) Quantification of Env (gp120) incorporation into virions normalised to Gag (p24) density, produced in Jurkat and primary CD4^+^ T cells. (**D**) Quantification of virus release as measured by Gag (p24) density normalised to total Gag (p24 + p55) produced in infected Jurkat cells. (**E**) Virus budding from infected Jurkat T cells and primary CD4^+^ T cells at 48 hpi measured by quantifying RT activity in viral supernatants by SG-PERT qPCR from infections shown in (**A**). (**F**) Quantification of infected Jurkat T cells at 24 hpi measured by flow cytometry analysis for the % Gag+ cells (left) and Gag MFI of the Gag+ population (middle). The percentage live cells for WT and W757A is shown (right). (**G**) Virus release from infected Jurkat T cells (from (**F**)) at 16 hpi (left) and 24 hpi (middle) measured by quantifying RT activity in viral supernatants by SG-PERT qPCR. Right panel shows combined data from both time points. Data show the mean and SEM from at least three independent experiments compared using a two-tailed unpaired *t*-test (ns, not significant; ** *p* < 0.01, *** *p* < 0.001 **** *p* < 0.0001).

Integrating the striking defect in cell–cell spread and VS formation with super-resolution single-molecule imaging of Env suggested that mutating W757 in the EnvCT perturbed Env mobility in the PM and therefore the temporal and spatial recruitment of Env to virus assembly sites; however, we were also struck by the observation that Gag also failed to polarise to the VS when cells were infected with the W757A Env mutant virus (Figure 1B). This suggests that perturbing W757 by alanine mutagenesis may negatively impact the regulation of infectious HIV-1 egress from T cells for subsequent cell–cell spread. To explore this mutant further, we next quantified the infectivity and the cell-free budding of virus produced by Jurkat T cells and primary CD4^+^ T cells infected with WT or W757A virus. Measuring virus infectivity by titrating virus-containing supernatants from infected T cells onto HeLa TZM-bl cells and normalising the relative light units (RLU) to reverse transcriptase (RT) units measured by SG-PERT assay showed viral particles harbouring the W757A mutant EnvCT were significantly less infectious than WT virus was (Figure 4A). Truncation of the majority of the EnvCT (CT∆144 virus) renders HIV-1 non-infectious and spread in T cell lines and primary CD4^+^ T cells due to an Env incorporation defect [10]. By contrast, examining Env in W757A virions by immunoblotting showed that incorporation of Env gp120 into viral particles was similar between WT and W757A virus (Figure 4B), with no significant difference between these viruses in the gp120:p24 ratio in virions (Figure 4C). The lack of an Env incorporation defect for W757A virus is consistent with the single-molecule tracking data showing that both WT Env and W757A Env get trapped in the Gag lattice at the PM, and thus would be expected to get incorporated into budding virions [48].

Interestingly, quantification of these western blots revealed that significantly less p24Gag was present in the purified virus supernatant relative to the amount of cell-associated Gagp24/p55 for W757A when compared to WT virus (Figure 4D). In addition, quantification of virus release by performing SG-PERT assays for reverse transcriptase activity in culture supernatants showed that infection of both Jurkat and primary CD4^+^ T cells with W757A virus resulted in a significant reduction in HIV-1 budding compared to WT virus (Figure 4E). Importantly, mutating W757 did not result in amino acid changes to Rev that have been reported to mediate budding defects when the EnvCT is truncated [49]. Exploring this further, we infected Jurkat T cells with VSV-G pseudotyped W757A and WT virus and performed additional analysis at earlier time points post-infection (Figure 4F,G). Flow cytometry analysis at 24 hpi showed a 3-fold decrease in the Gag MFI of W757A-infected T cells compared to WT-infected cells (Figure 4F), but a more pronounced and significant 7-fold decrease in HIV-1 budding at both 16 hpi and 24 hpi post-infection (Figure 4G), time points that correspond to a single round of HIV-1 replication. Of note, truncation of the HIV-1 EnvCT (ΔCT) in the context of an otherwise full-length NL4.3 virus [34] did not reduce virus budding from HIV-1-infected Jurkat T cells, nor virus release from 293T cells transfected with virus plasmids (Supplementary Figure S3). Specifically, we observed equivalent levels of virus in cell culture supernatants for WT and ΔCT virus, by contrast to the W757A, where budding was significantly reduced from both cell types (Supplementary Figure S3). Taken together, these data suggest that the specific mutation of W757 by replacing a tryptophan with an alanine in the context of the full-length EnvCT appears to negatively impact virion assembly and/or egress from T cells.

### 3.4. Synthesis, Processing, and Recycling of EnvCT W757A Is Equivalent to That of WT EnvCT

**Figure 5:**
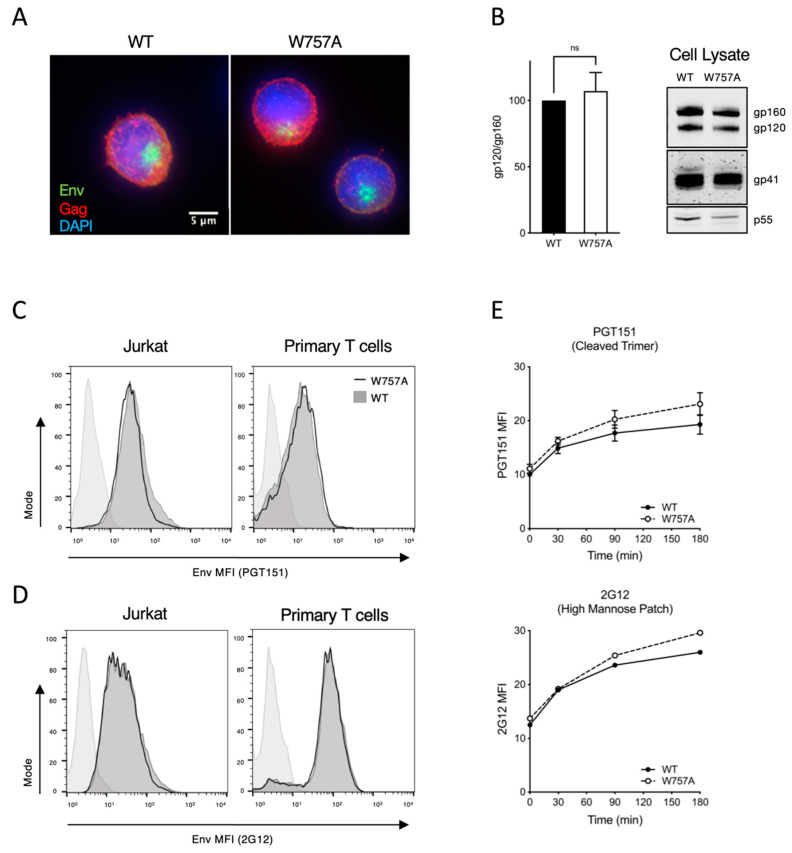
Synthesis, processing and recycling of EnvCT W757A is equivalent to that of WT EnvCT. (**A**) Intracellular immunofluorescence staining of Env (green) and Gag (red) in Jurkat cells infected with WT and W757A at 48 hpi. (**B**) Western blot of infected Jurkat cell lysates harvested 48 hpi and probed with anti-gp120, anti-gp41 and anti-Gag p55/p24. Quantification of Env processing (gp120/gp160). Data show the mean and SEM from at least three independent experiments compared using a two-tailed paired *t*-test (ns, not significant). (**C**) Flow cytometry analysis of cell surface Env levels on cells infected with WT (dark grey, filled) and W757A (solid black line, unfilled) virus measured using bNAb PGT151. Uninfected control is shown as light grey-filled histogram. (**D**) Flow cytometry analysis of cell surface Env levels on cells infected with WT (dark grey, filled) and W757A (solid black line, unfilled) virus measured using 2G12 as in (**C**). (**E**) Time course of endocytosed Env recycling back to the plasma membrane measured by flow cytometry. Env was stained with either PGT151 or 2G12. Envelope recycling was measured by staining with envelope antibody and a PE-secondary on ice followed by Cy5 at 37 °C. Recycling is shown as the MFI of PE^+^CY5^+^ signal on the surface over time. Data show the mean and SEM from three independent experiments.

**Figure 6:**
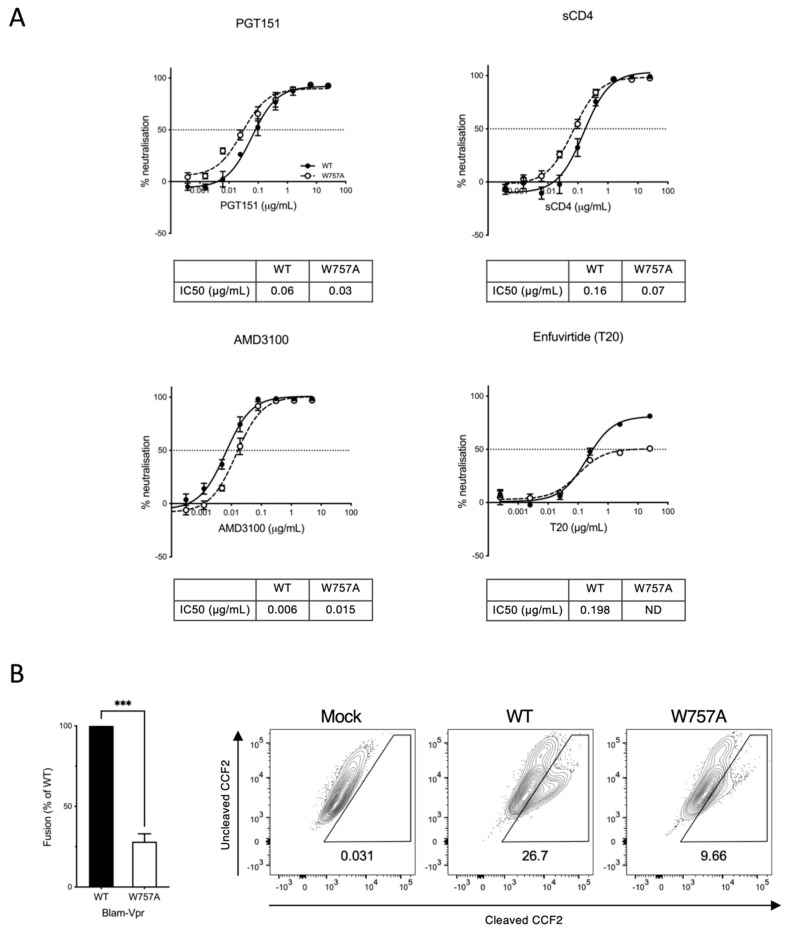
W757A virus shows a defect in viral fusion. (**A**) Neutralisation of WT and W757A virus (produced from Jurkat T cells) by PGT151 bNAb, sCD4, AMD3100 and T20 by neutralisation assay using HeLa TZM-bl cells. IC_50_ values calculated by nonlinear regression analysis of the neutralisation curves. ND denotes not determined. (**B**) W757A Env is defective in viral fusion compared to WT virus as measured by the BlaM-Vpr fusion assay. Data are the mean and SEM compared using an unpaired *t*-test (*** *p* < 0.001). Mock refers to co-culture between uninfected T cells and target T cells.

To address the observation that W757 in the EnvCT is required for viral infectivity, but apparently not virion incorporation, we tested Env expression, processing, localisation and trafficking in infected T cells. VS formation (and subsequent cell–cell spread) is dependent on expression of the Env glycoprotein on the surface of infected cells interacting with cell surface receptors on target cells [27]. Immunofluorescence microscopy showed no difference in intracellular staining of WT and W757A Env (Figure 5A), with both displaying perinuclear localisation consistent with the bulk of Env residing in Golgi compartments as expected [15,20]. Immunoblotting of infected cell lysates confirmed similar levels of unprocessed Env gp160 and the furin-cleavage products gp120 and gp41 (Figure 5B). To compare surface expression of Env on infected T cells, flow cytometry analysis was performed. WT- and W757A-infected T cells (Jurkat and primary CD4^+^ T cells) expressed identical levels of Env on the surface of infected cells when stained with either the Env-specific mAbs PGT151 (for the gp120:gp41 interface and functional timers) (Figure 5C) or 2G12 (recognising a non-conformation-dependent carbohydrate epitope on gp120) (Figure 5D). Once at the PM, a highly conserved membrane-proximal AP-2 binding motif (YxxL) acts to limit the amount of Env presented at the surface of infected cells with internalised Env being recycled back to the PM [13,14,15,16,17,20,39]. To test the recycling kinetics of WT and W757A Env, we used a dual-fluorophore flow cytometry-based assay [39,50]. Two Env antibodies, 2G12 and PGT151, were allowed to bind surface Env at 4 °C and detected using a PE-conjugated secondary antibody. Recycling over time was allowed to proceed at 37 °C, and the population of Env that returned to the surface was detected with a second Cy5-secondary, resulting in double PE/Cy5-labelled Env. No significant difference in recycling of endocytosed Env back to the PM was seen between WT and W757A Env (Figure 5E). Finally, we tested whether Env W757A was competent for CD4 and co-receptor (CXCR4) binding and trimer formation using neutralisation assays [40]. WT and W757A viruses were both neutralised by bNAb PGT151 (that binds to the gp120/gp41 trimer interface and detects functional Env trimers [41,51]), soluble CD4 (sCD4) and the CXCR4 co-receptor inhibitor AMD3100 [52] (Figure 6A). While subtle differences in neutralisation were observed between WT and W757A virus, these are insufficient to explain the striking defect in viral infectivity. In order to investigate whether a fusion defect may be responsible for the decrease in Env W757A infectivity, neutralisation with the fusion inhibitor Enfuvirtide (T20) was carried out. Strikingly Env W757A was unable to be completely neutralised with T20, compared to WT Env, where a maximum inhibition of 50% was achieved (Figure 6A). To test whether W757 was fusion-competent, the BlaM-Vpr fusion assay was used to measure the capacity of Env W757A virions to fuse with target cells, resulting in BlaM-Vpr release into the target cell and cleavage of CCF2 [37]. Figure 6B shows that Env W757A was significantly less able to mediate virion fusion compared to WT virus, demonstrating that the infectivity defect of W757A can be explained by a defect in fusion of the viral and host cell membranes and thus reduced virus entry.

### 3.5. Conservation of W757 across Different HIV Lineages and Ancestors

**Figure 7:**
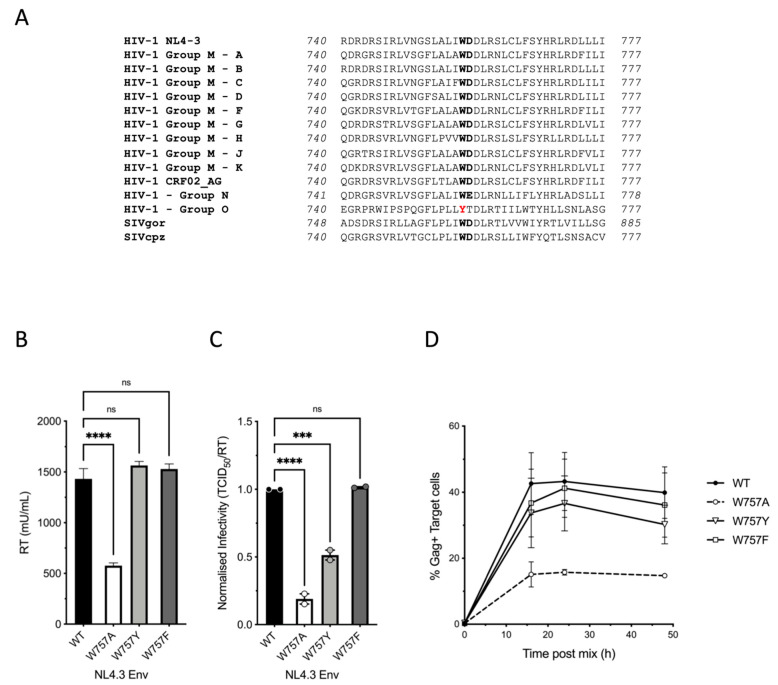
Conservation of W757 across HIV-1 and SIV lineages. (**A**) Amino acid alignment of the LLP2 domain of the EnvCT from different HIV and SIV consensus sequences. See also Supplementary Figure S4. (**B**) Viral budding, (**C**) infectivity, and (**D**) cell–cell spread can be rescued by substitution at position 757 with either tyrosine Y, or phenylalanine, F. Data are the mean and SEM compared using a two-tailed paired *t*-test (ns, not significant; *** *p* < 0.001, **** *p* < 0.0001).

Finally, we sought to explore conservation of W757 across HIV-1 groups and related lentiviruses. Examining 194 Env sequences at position 757 across HIV-1 groups M, N, O, and P, SIVgor and SIVcpz using Chromaclade [45,53] illustrated that a W at position 757 is highly conserved amongst HIV-1 group M, SIVcpz and HIV-1 group N; but not group O, or HIV-2 and the related SIVsmm and SIVmac viruses (Figure 7A and Supplementary Figure S4). Noting that all HIV-1 sequences (M, N, O and P) contained an aromatic residue at 757 (W or Y), we hypothesised that conservation of an aromatic residue at this position was important and that substituting the A for a Y or F would rescue virus replication and spread. Indeed, substituting the A at position 757 with a Y (HIV-1 O group) or F (another aromatic) rescued the W757 mutant and phenocopied the WT virus, restoring viral budding (Figure 7B), infectious virus production (Figure 7C) and cell–cell spread (Figure 7D). Taken together, this data implicate evolutionary conservation of an aromatic residue in the EnvCT at position 757 as a potentially important feature of HIV-1.

## 4. Discussion

**Figure 8:**
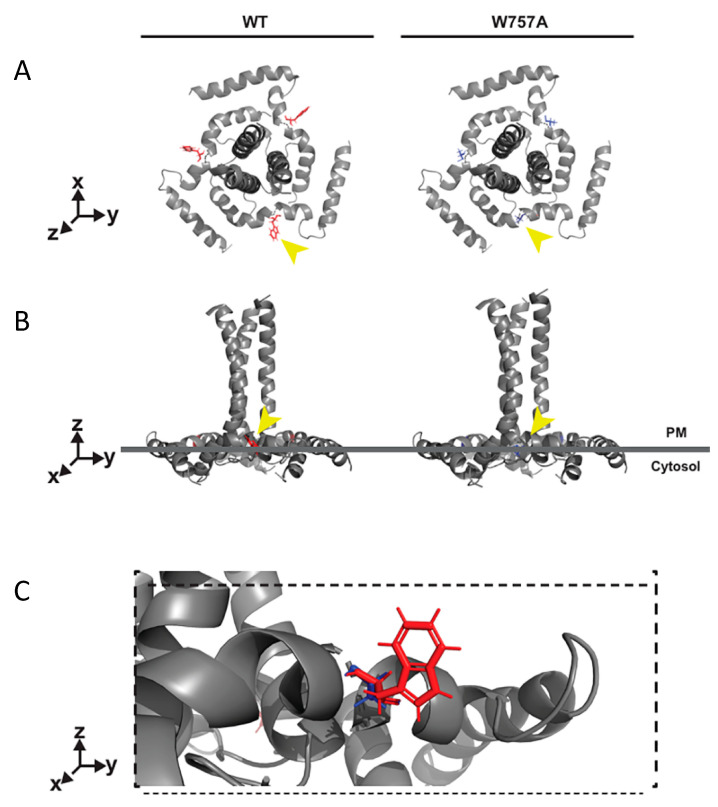
W757 within the EnvCT LLP2 helix may stabilise the EnvCT baseplate. (**A**) Top-down and (**B**) side-on models of trimeric EnvCT and TMD within the plasma membrane. Tryptophan (red) and alanine (blue) are highlighted in (**C**). The aromatic side chain of W757 (indicated by the yellow arrow) is predicted to be buried into the membrane, maintaining the EnvCT baseplate conformation, whereas alanine substitution at this position may allow for more conformational flexibility in the quaternary structure of the EnvCT baseplate, disrupting the TMD and perhaps MPER/gp120 conformations.

Here, we have uncovered a key role for a highly conserved tryptophan residue (W) in the HIV-1 EnvCT that is essential for correct temporal and spatial regulation of infectious HIV-1 assembly following cell–cell contact and subsequent viral spread between T cells. The mechanism by which HIV-1 assembly is regulated, and the spatiotemporal events governing Env and Gag recruitment to the contact zone during synapse formation leading to polarised viral assembly, remains largely unclear. Moreover, why lentiviruses conserve such an unusually long EnvCT and what role it plays on HIV-1 assembly and infectivity remain somewhat enigmatic. Given the high efficiency of cell–cell spread and its contribution to viral dissemination, understanding how this process is coordinated is important [27,28,29]. Multiple (but not mutually exclusive) mechanisms for Env and Gag recruitment to sites of virus assembly at the VS have been proposed. In the case of Env, there is evidence for Env being directed to the VS via lateral diffusion, outward transport from polarised secretory apparatus and recycling [22,24,54]. By contrast, Gag is thought to be recruited independently of Env, with evidence for lateral diffusion [31] and polarised trafficking [55] to the contact zone. By coupling viral assays with single-particle tracking at super-resolved virus assembly sites we find that the EnvCT via W757 is required to direct Env and Gag to sites of cell–cell contact and mediate VS formation and spreading infection in T cells. Notably, single-molecule tracking revealed that mutating W757 resulted in Env diffusing more freely within the PM compared to WT Env. Structural insights of the EnvCT by solution-state NMR revealed that much of the EnvCT associates closely with cellular membranes, and W757 at the start of the first alpha helix (LLP-2) in the EnvCT appears to provide a crucial anchor point in order to regulate membrane diffusion (Figure 8) [12,56]. Perturbation of this W757 anchor point may lead to changes in the structure of the putative LLP-2 baseplate and could explain an increase in PM diffusivity of the W757A mutant Env trimer compared to the native EnvCT [56]. Disruption of the EnvCT baseplate by W757A may not just affect membrane diffusion, but could also alter interactions of the EnvCT with host cell proteins or underlying cytoskeleton machinery that are required for polarised recruitment of Env to the VS [27]. The increased diffusion of W757A Env provides a plausible explanation for decreased W757A Env at the VS and suggests that the polarisation of Env to sites of cell–cell contact is not mediated simply by immobilising surface-associated Env following binding to CD4 on target cells, but rather that cell–cell contact must specifically trigger or signal Env recruitment to the contact zone. Consistent with this notion, W757A was competent for Env-CD4 and co-receptor binding by neutralisation assay. We also found that the mobility of this mutant at sites of virus assembly was unperturbed when compared to WT Env trimers, suggesting that conformational changes in the baseplate structure or membrane association of LLP-2 do not impact Env incorporation and retention in the Gag lattice. This is consistent with our biochemical observations that W757A showed no incorporation defect, demonstrating that once Env associates with the Gag lattice it will be incorporated into viral particles. Of note, while our data indicate that the earliest events of VS formation and cell–cell assembly of HIV-1 require correct and regulated Env movement within the plasma membrane, they do not exclude that subsequent Env recruitment may be mediated by additional mechanisms described above.

Env is known to be a key driver of VS formation and cell–cell spread, and inhibiting Env–receptor interactions abolishes VS formation [27]. We now show that the contribution of Env during VS formation is more than mediating stable cell–cell interactions and providing adhesive interactions, rather the EnvCT appears to signal in some way to regulate the recruitment of Gag to the VS, thus to control the timing and location of virus assembly. Mechanistically, these observations may be explained by two possibilities. Firstly, they may allude to a sequence of assembly events in which the EnvCT in the PM initiates Gag lattice formation and budding following contact with a susceptible target cell [31]. A second possibility is that EnvCT at the neck of the budding virions is essential to complete the budding process [4] and that mutating a key residue the EnvCT inhibits this; however, while this may explain the defect in viral spread, it does not explain the dramatic lack of Gag polarisation to the VS The initial engagement of Env and CD4/co-receptor results in polarisation of the cellular cytoskeleton to the contact site, and disrupting the actin cytoskeleton inhibits VS formation as well as plasma membrane virus assembly platforms [27,28,32,57]. Whether the underlying host cell cytoskeletal or associated cellular proteins link the EnvCT to polarised Gag lattice formation in some way (via undefined host factors) is an interesting possibility. It cannot be excluded that direct interactions between the EnvCT and Gag take place; however, while evidence for direct interactions have been shown biochemically in vitro [58,59], they have not been convincingly demonstrated consistently, and we were unable to replicate this during the course of this study. While it was initially surprising that the W757A mutation negatively impacted on HIV-1 budding, we consistently observed this when employing a range of measurements using both Jurkat and primary CD4^+^ T cells, whereas truncation of the EnvCT showed no budding defect. We interpret these data to mean that when the EnvCT is present, there is a requirement for an aromatic residue at position 757 in order for viral budding to proceed efficiently, but that deletion of the majority of the EnvCT bypasses this and allows for Gag-mediated HIV-1 budding. Of note, the EnvCT YxxL mutant has been reported to affect the site of virus budding in polarised epithelial cells and T cells [23,60], supportive of a role for the EnvCT in regulating viral assembly and budding. Our finding that viral budding is perturbed following alanine substitution at W757 would be consistent with the EnvCT playing some regulatory role in HIV-1 budding, with the caveat that it has been previously shown that almost complete EnvCT truncation is tolerated in the context of Gag-driven budding, as we have also shown here. Thus, the requirement for conserved motifs and the ability of HIV-1 to tolerate variation in the EnvCT is likely nuanced, rather than binary. Clearly, further work is needed to determine the molecular interactions that allow the EnvCT to regulate the later stages of viral egress, particularly during cell–cell spread.

In addition to a lack of VS formation, cell–cell spread was also affected by a defect in infectivity of the W757A mutant virus. This was initially surprising, given that many other features of Env W757A remained unchanged, and unlike EnvCT truncation (CT∆144) that also abrogates cell–cell spread [1,10,49], EnvCT W757A had no incorporation defect and did not display altered cell surface expression or recycling in T cell lines. Further analysis revealed that this infectivity defect was explained by the W757A virus showing a striking defect in fusion and thus viral entry, indicating that a single residue change in LLP-2 distal to the fusion peptide significantly impacted fusion domain structure and function. In support of our data, it has been reported that the LLP-2 helix influences fusogenicity and that viruses containing EnvCT truncations are less sensitive to T20 inhibition [61,62,63]. Recent structural insights of the EnvCT have identified the role of the LLP-2 helix to act as a baseplate that supports the Env transmembrane domain (TMD) and stabilises the MPER region of Env, holding both in the correct structural antigenic state [56]. It is highly plausible that W757 in LLP-2 is key to maintenance of the EnvCT baseplate (Figure 8). Mutation of the hydrophobic W757 may be sufficient to perturb the baseplate, conferring conformational changes via the TMD [56] on the MPER and Env fusion domains, destabilising the Env ectodomain, leading to reduced Env fusogenicity. Thus, our data support the notion that the EnvCT baseplate plays a putative role in regulating viral entry [56]. Of note, many retroviruses that contain shorter EnvCTs, such as MLV, exploit a C-terminal R peptide that controls membrane fusion activity [64], while HIV-1 EnvCT truncation is reported to impact on HIV Env ectodomain fusion activity [16,65]. Thus, our data are consistent with a key function of the long lentiviral EnvCT and its membrane-associated structure in regulating fusion, and in the case of HIV-1, this is via LLP-2 and specifically, residue W757. How HIV-2 and SIVs regulate these functions warrants further study, not least because HIV-2 does not appear to have the same dependence on the EnvCT for replication in T cells [35].

In conclusion, here we have identified a single tryptophan residue in HIV-1 EnvCT that potently regulates HIV-1 assembly at the VS, viral fusion and spread of HIV-1 between T cells. That this W757 residue is highly conserved in pandemic HIV-1 group M and its SIVcpz ancestor suggests a fundamental role for conservation of this reside in HIV-1 replication.

## Data Availability

Not applicable.

## Supplementary Materials

The following are available online at https://www.mdpi.com/article/10.3390/v14010129/s1, Figure S1: EnvCT W757A is defective in T cell–T cell spread. Figure S2: WT and W757A mutant Env diffusion coefficient (*D_apparent_*) and *S_MSS_* histograms for tracks proximal and distal to Gag lattice assembly sites. Figure S3: Budding of Env ∆CT in Jurkat and 293T cells. Figure S4: Conservation of W757 among HIV and SIV envelope glycoproteins3.

## References

[1] Checkley M.A., Luttge B.G., Freed E.O. (2011). HIV-1 envelope glycoprotein biosynthesis, trafficking, and incorporation. J. Mol. Biol..

[2] Ono A., Ablan S.D., Lockett S.J., Nagashima K., Freed E.O. (2004). Phosphatidylinositol (4,5) bisphosphate regulates HIV-1 Gag targeting to the plasma membrane. Proc. Natl. Acad. Sci. USA.

[3] Alfadhli A., Barklis R.L., Barklis E. (2009). HIV-1 matrix organizes as a hexamer of trimers on membranes containing phosphatidylinositol-(4,5)-bisphosphate. Virology.

[4] Buttler C.A., Pezeshkian N., Fernandez M.V., Aaron J., Norman S., Freed E.O., Van Engelenburg S.B. (2018). Single molecule fate of HIV-1 envelope reveals late-stage viral lattice incorporation. Nat. Commun..

[5] Stuchell M.D., Garrus J.E., Müller B., Stray K.M., Ghaffarian S., McKinnon R., Kräusslich H.G., Morham S.G., Sundquist W.I. (2004). The human endosomal sorting complex required for transport (ESCRT-I) and its role in HIV-1 budding. J. Biol. Chem..

[6] Morita E., Sandrin V., McCullough J., Katsuyama A., Baci Hamilton I., Sundquist W.I. (2011). ESCRT-III protein requirements for HIV-1 budding. Cell Host Microbe.

[7] Prescher J., Baumgärtel V., Ivanchenko S., Torrano A.A., Bräuchle C., Müller B., Lamb D.C. (2015). Super-Resolution Imaging of ESCRT-Proteins at HIV-1 Assembly Sites. PLoS Pathog..

[8] Van Engelenburg S.B., Shtengel G., Sengupta P., Waki K., Jarnik M., Ablan S.D., Freed E.O., Hess H.F., Lippincott-Schwartz J. (2014). Distribution of ESCRT Machinery at HIV Assembly Sites Reveals Virus Scaffolding of ESCRT Subunits. Science.

[9] Murphy R.E., Saad J.S. (2020). The interplay between HIV-1 gag binding to the plasma membrane and env incorporation. Viruses.

[10] Murakami T., Freed E.O. (2000). The long cytoplasmic tail of gp41 is required in a cell type-dependent manner for HIV-1 envelope glycoprotein incorporation into virions. Proc. Natl. Acad. Sci. USA.

[11] Postler T.S., Desrosiers R.C. (2013). The Tale of the Long Tail: The Cytoplasmic Domain of HIV-1 gp41. J. Virol..

[12] Murphy R.E., Samal A.B., Vlach J., Saad J.S. (2017). Solution Structure and Membrane Interaction of the Cytoplasmic Tail of HIV-1 gp41 Protein. Structure.

[13] Berlioz-Torrent C., Shacklett B.L., Erdtmann L., Delamarre L., Bouchaert I., Sonigo P., Dokhelar M.C., Benarous R. (1999). Interactions of the Cytoplasmic Domains of Human and Simian Retroviral Transmembrane Proteins with Components of the Clathrin Adaptor Complexes Modulate Intracellular and Cell Surface Expression of Envelope Glycoproteins. J. Virol..

[14] Ohno H., Aguilar R.C., Fournier M.C., Hennecke S., Cosson P., Bonifacino J.S. (1997). Interaction of endocytic signals from the HIV-1 envelope glycoprotein complex with members of the adaptor medium chain family. Virology.

[15] Byland R., Vance P.J., Hoxie J.A., Marsh M. (2007). A conserved dileucine motif mediates clathrin and AP-2-dependent endocytosis of the HIV-1 envelope protein. Mol. Biol. Cell.

[16] Wyss S., Berlioz-Torrent C., Boge M., Blot G., Höning S., Benarous R., Thali M. (2001). The Highly Conserved C-Terminal Dileucine Motif in the Cytosolic Domain of the Human Immunodeficiency Virus Type 1 Envelope Glycoprotein Is Critical for Its Association with the AP-1 Clathrin Adapter. J. Virol..

[17] Bhakta S.J., Shang L., Prince J.L., Claiborne D.T., Hunter E. (2011). Mutagenesis of tyrosine and di-leucine motifs in the HIV-1 envelope cytoplasmic domain results in a loss of Env-mediated fusion and infectivity. Retrovirology.

[18] Stano A., Leaman D.P., Kim A.S., Zhang L., Autin L., Ingale J., Gift S.K., Truong J., Wyatt R.T., Olson A.J. (2017). Dense Array of Spikes on HIV-1 Virion Particles. J. Virol..

[19] Rusert P., Kouyos R.D., Kadelka C., Ebner H., Schanz M., Huber M., Braun D.L., Hozé N., Scherrer A., Magnus C. (2016). Determinants of HIV-1 broadly neutralizing antibody induction. Nat. Med..

[20] Groppelli E., Len A.C., Granger L.A., Jolly C. (2014). Retromer Regulates HIV-1 Envelope Glycoprotein Trafficking and Incorporation into Virions. PLoS Pathog..

[21] Qi M., Williams J.A., Chu H., Chen X., Wang J.J., Ding L., Akhirome E., Wen X., Lapierre L.A., Goldenring J.R. (2013). Rab11-FIP1C and Rab14 Direct Plasma Membrane Sorting and Particle Incorporation of the HIV-1 Envelope Glycoprotein Complex. PLoS Pathog..

[22] Wang L., Sandmeyer A., Hübner W., Li H., Huser T., Chen B.K. (2020). Recruitment of Env to the HIV-1 T cell virological synapse by targeted and 2 sustained Env recycling. bioRxiv.

[23] Deschambeault J., Lalonde J.-P., Cervantes-Acosta G., Lodge R., Cohen É.A., Lemay G. (1999). Polarized Human Immunodeficiency Virus Budding in Lymphocytes Involves a Tyrosine-Based Signal and Favors Cell-to-Cell Viral Transmission. J. Virol..

[24] Kirschman J., Qi M., Ding L., Hammonds J., Dienger-Stambaugh K., Wang J.-J., Lapierre L.A., Goldenring J.R., Spearman P. (2017). HIV-1 Envelope Glycoprotein Trafficking through the Endosomal Recycling Compartment Is Required for Particle Incorporation. J. Virol..

[25] Sattentau Q. (2008). Avoiding the void: Cell-to-cell spread of human viruses. Nat. Rev. Microbiol..

[26] Jolly C., Mitar I., Sattentau Q.J. (2007). Adhesion Molecule Interactions Facilitate Human Immunodeficiency Virus Type 1-Induced Virological Synapse Formation between T Cells. J. Virol..

[27] Jolly C., Kashefi K., Hollinshead M., Sattentau Q.J. (2004). HIV-1 Cell to Cell Transfer across an Env-induced, Actin-dependent Synapse. J. Exp. Med..

[28] Chen P., Hübner W., Spinelli M.A., Chen B.K. (2007). Predominant Mode of Human Immunodeficiency Virus Transfer between T Cells Is Mediated by Sustained Env-Dependent Neutralization-Resistant Virological Synapses. J. Virol..

[29] Sourisseau M., Sol-Foulon N., Porrot F., Blanchet F., Schwartz O. (2007). Inefficient Human Immunodeficiency Virus Replication in Mobile Lymphocytes. J. Virol..

[30] Murooka T.T., Deruaz M., Marangoni F., Vrbanac V.D., Seung E., Von Andrian U.H., Tager A.M., Luster A.D., Mempel T.R. (2012). HIV-infected T cells are migratory vehicles for viral dissemination. Nature.

[31] Hübner W., McNerney G.P., Chen P., Dale B.M., Gordon R.E., Chuang F.Y.S., Li X.D., Asmuth D.M., Huser T., Chen B.K. (2009). Quantitative 3D video microscopy of HIV transfer across T cell virological synapses. Science.

[32] Rudnicka D., Feldmann J., Porrot F., Wietgrefe S., Guadagnini S., Prévost M.-C., Estaquier J., Haase A.T., Sol-Foulon N., Schwartz O. (2009). Simultaneous Cell-to-Cell Transmission of Human Immunodeficiency Virus to Multiple Targets through Polysynapses. J. Virol..

[33] Tremblay M., Rooke R., Geleziunas R., Wainberg M.A., Sullivan A.K., Tsoukas C., Gilmore N., Shematek G. (1989). New cd4(+) cell line susceptible to infection by hiv-1. J. Med. Virol..

[34] Haider T., Snetkov X., Jolly C. (2021). HIV envelope tail truncation confers resistance to SERINC5 restriction. Proc. Natl. Acad. Sci. USA.

[35] Groves N.S., Bruns M.M., van Engelenburg S.B. (2020). A quantitative live-cell superresolution imaging framework for measuring the mobility of single molecules at sites of virus assembly. Pathogens.

[36] Pizzato M., Erlwein O., Bonsall D., Kaye S., Muir D., McClure M.O. (2009). A one-step SYBR Green I-based product-enhanced reverse transcriptase assay for the quantitation of retroviruses in cell culture supernatants. J. Virol. Methods.

[37] Cavrois M., De Noronha C., Greene W.C. (2002). A sensitive and specific enzyme-based assay detecting HIV-1 virion fusion in primary T lymphocytes. Nat. Biotechnol..

[38] Schindelin J., Arganda-Carreras I., Frise E., Kaynig V., Longair M., Pietzsch T., Preibisch S., Rueden C., Saalfeld S., Schmid B. (2012). Fiji: An open-source platform for biological-image analysis. Nat. Methods.

[39] Mesner D., Hotter D., Kirchhoff F., Jolly C. (2020). Loss of Nef-mediated CD3 down-regulation in the HIV-1 lineage increases viral infectivity and spread. Proc. Natl. Acad. Sci. USA.

[40] Montefiori D.C. (2009). Measuring HIV neutralization in a luciferase reporter gene assay. Methods Mol. Biol..

[41] Falkowska E., Le K.M., Ramos A., Doores K.J., Lee J.H., Blattner C., Ramirez A., Derking R., vanGils M.J., Liang C.H. (2014). Broadly neutralizing HIV antibodies define a glycan-dependent epitope on the prefusion conformation of gp41 on cleaved envelope trimers. Immunity.

[42] Salzwedel K., West J.T., Hunter E. (1999). A Conserved Tryptophan-Rich Motif in the Membrane-Proximal Region of the Human Immunodeficiency Virus Type 1 gp41 Ectodomain Is Important for Env-Mediated Fusion and Virus Infectivity. J. Virol..

[43] Muñoz-Barroso I., Salzwedel K., Hunter E., Blumenthal R. (1999). Role of the Membrane-Proximal Domain in the Initial Stages of Human Immunodeficiency Virus Type 1 Envelope Glycoprotein-Mediated Membrane Fusion. J. Virol..

[44] Crooks G.E., Hon G., Chandonia J.M., Brenner S.E. (2004). WebLogo: A sequence logo generator. Genome Res..

[45] Foley B., Korber B., Leitner T., Apetrei C., Hahn B., Mizrachi I., Mullins J.I., Rambaut A., Wolinsky S. (2018). HIV Sequence Compendium 2018.

[46] Lambelé M., Labrosse B., Roch E., Moreau A., Verrier B., Barin F., Roingeard P., Mammano F., Brand D. (2007). Impact of Natural Polymorphism within the gp41 Cytoplasmic Tail of Human Immunodeficiency Virus Type 1 on the Intracellular Distribution of Envelope Glycoproteins and Viral Assembly. J. Virol..

[47] Blot G., Janvier K., Le Panse S., Benarous R., Berlioz-Torrent C. (2003). Targeting of the Human Immunodeficiency Virus Type 1 Envelope to the trans-Golgi Network through Binding to TIP47 Is Required for Env Incorporation into Virions and Infectivity. J. Virol..

[48] Pezeshkian N., Groves N.S., van Engelenburg S.B. (2019). Single-molecule imaging of HIV-1 envelope glycoprotein dynamics and Gag lattice association exposes determinants responsible for virus incorporation. Proc. Natl. Acad. Sci. USA.

[49] Durham N.D., Chen B.K. (2015). HIV-1 Cell-Free and Cell-to-Cell Infections Are Differentially Regulated by Distinct Determinants in the Env gp41 Cytoplasmic Tail. J. Virol..

[50] Anand S.P., Grover J.R., Tolbert W.D., Prévost J., Richard J., Ding S., Baril S., Medjahed H., Evans D.T., Pazgier M. (2019). Antibody-Induced Internalization of HIV-1 Env Proteins Limits Surface Expression of the Closed Conformation of Env. J. Virol..

[51] Blattner C., Lee J.H., Sliepen K., Derking R., Falkowska E., delaPeña A.T., Cupo A., Julien J.P., vanGils M., Lee P.S. (2014). Structural delineation of a quaternary, cleavage-dependent epitope at the gp41-gp120 interface on intact HIV-1 env trimers. Immunity.

[52] Donzella G.A., Schols D., Lin S.W., Esté J.A., Nagashima K.A., Maddon P.J., Allaway G.P., Sakmar T.P., Henson G., De Clercq E. (1998). AMD3100, a small molecule inhibitor of HIV-1 entry via the CXCR4 co- receptor. Nat. Med..

[53] Monit C., Goldstein R.A., Towers G.J. (2019). ChromaClade: Combined visualisation of phylogenetic and sequence data. BMC Evol. Biol..

[54] Starling S., Jolly C. (2016). LFA-1 Engagement Triggers T Cell Polarization at the HIV-1 Virological Synapse. J. Virol..

[55] Groppelli E., Starling S., Jolly C. (2015). Contact-Induced Mitochondrial Polarization Supports HIV-1 Virological Synapse Formation. J. Virol..

[56] Piai A., Fu Q., Cai Y., Ghantous F., Xiao T., Shaik M.M., Peng H., Rits-Volloch S., Chen W., Seaman M.S. (2020). Structural basis of transmembrane coupling of the HIV-1 envelope glycoprotein. Nat. Commun..

[57] Jolly C., Mitar I., Sattentau Q.J. (2007). Requirement for an Intact T-Cell Actin and Tubulin Cytoskeleton for Efficient Assembly and Spread of Human Immunodeficiency Virus Type 1. J. Virol..

[58] Cosson P. (1996). Direct interaction between the envelope and matrix proteins of HIV-1. EMBO J..

[59] Alfadhli A., Staubus A.O., Tedbury P.R., Novikova M., Freed E.O., Barklis E. (2019). Analysis of HIV-1 Matrix-Envelope Cytoplasmic Tail Interactions. J. Virol..

[60] Lodge R., Göttlinger H., Gabuzda D., Cohen E.A., Lemay G. (1994). The intracytoplasmic domain of gp41 mediates polarized budding of human immunodeficiency virus type 1 in MDCK cells. J. Virol..

[61] Wyss S., Dimitrov A.S., Baribaud F., Edwards T.G., Blumenthal R., Hoxie J.A. (2005). Regulation of Human Immunodeficiency Virus Type 1 Envelope Glycoprotein Fusion by a Membrane-Interactive Domain in the gp41 Cytoplasmic Tail. J. Virol..

[62] Kalia V., Sarkar S., Gupta P., Montelaro R.C. (2003). Rational Site-Directed Mutations of the LLP-1 and LLP-2 Lentivirus Lytic Peptide Domains in the Intracytoplasmic Tail of Human Immunodeficiency Virus Type 1 gp41 Indicate Common Functions in Cell-Cell Fusion but Distinct Roles in Virion Envelope Incorpora. J. Virol..

[63] Lu L., Zhu Y., Huang J., Chen X., Yang H., Jiang S., Chen Y.H. (2008). Surface exposure of the HIV-1 Env cytoplasmic tail LLP2 domain during the membrane fusion process: Interaction with gp41 fusion core. J. Biol. Chem..

[64] Kubo Y., Tominaga C., Yoshii H., Kamiyama H., Mitani C., Amanuma H., Yamamoto N. (2007). Characterization of R peptide of murine leukemia virus envelope glycoproteins in syncytium formation and entry. Arch. Virol..

[65] Abrahamyan L.G., Mkrtchyan S.R., Binley J., Lu M., Melikyan G.B., Cohen F.S. (2005). The cytoplasmic tail slows the folding of human immunodeficiency virus type 1 Env from a late prebundle configuration into the six-helix bundle. J. Virol..

