# Supplementary material for: A Conserved Tryptophan in the Envelope Cytoplasmic Tail Regulates HIV-1 Assembly and Spread
Source: Viruses. 2022 Jan 12;14(1):129. doi: 10.3390/v14010129 (PMC8778169; doi:10.3390/v14010129)
Supplement: Supplementary file 1 [file viruses-14-00129-s001.zip › viruses-1544457-supplementary.pdf]

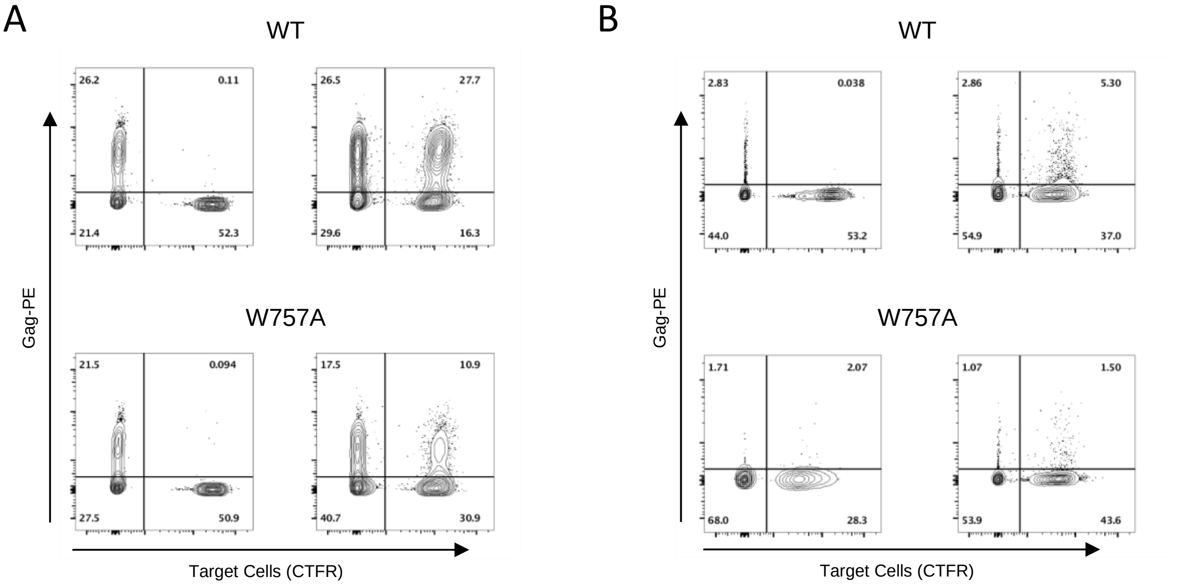

Supplementary Figure S1. EnvCT W757A is defective in T cell – T cell spread. Representative flow cytometry data of cell-cell spread between Jurkat T cells **(a)** and primary CD4^{+} T cells **(b)**. The percentage of cells in each quadrant is indicted. Related to Figure 1.

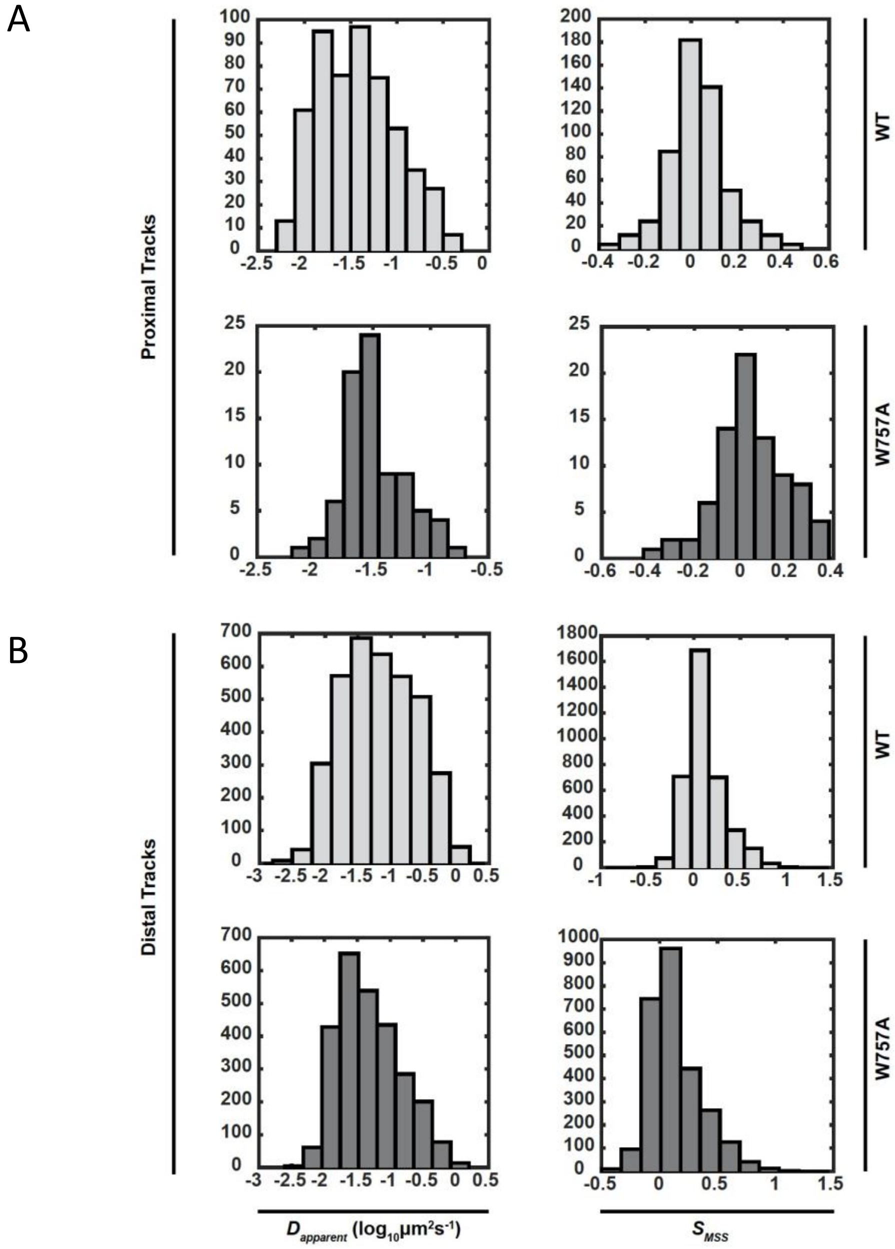

Supplementary Figure S2. WT and W757A mutant Env diffusion coefficient ($D_{apparent}$) and S_{MSS} histograms for tracks proximal **(a)** and distal **(b)** to Gag lattice assembly sites. Related to Figure 3.

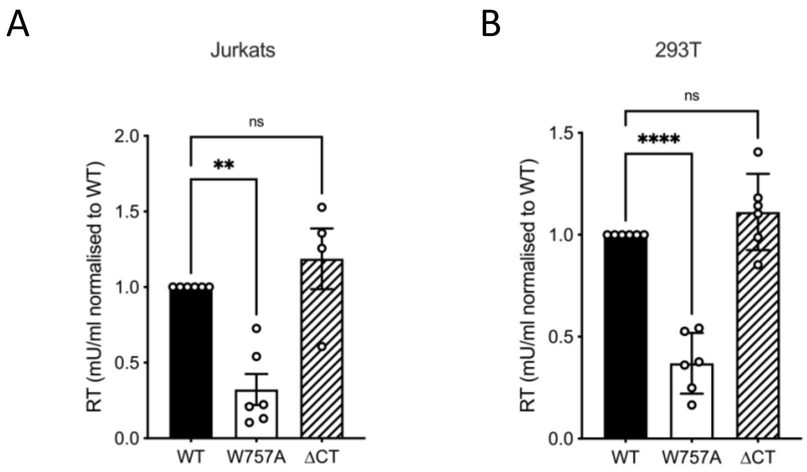

Supplementary Figure S3. **(a)** Virus release from Jurkat cells infected with VSV-G pseudotyped NL4.3 viruses at 24hpi, measured by quantifying RT activity in viral supernatants by SG-PERT assay. **(b)** Virus release from 293T cells transfected with NL4.3 viruses at 48hp post-transfection, measured by quantifying RT activity in viral supernatants by SG-PERT assay. Data show the mean and SEM from at least three independent experiments (ns, not significant; ** $p < 0.01$, **** $p < 0.0001$). Related to Figure 4.

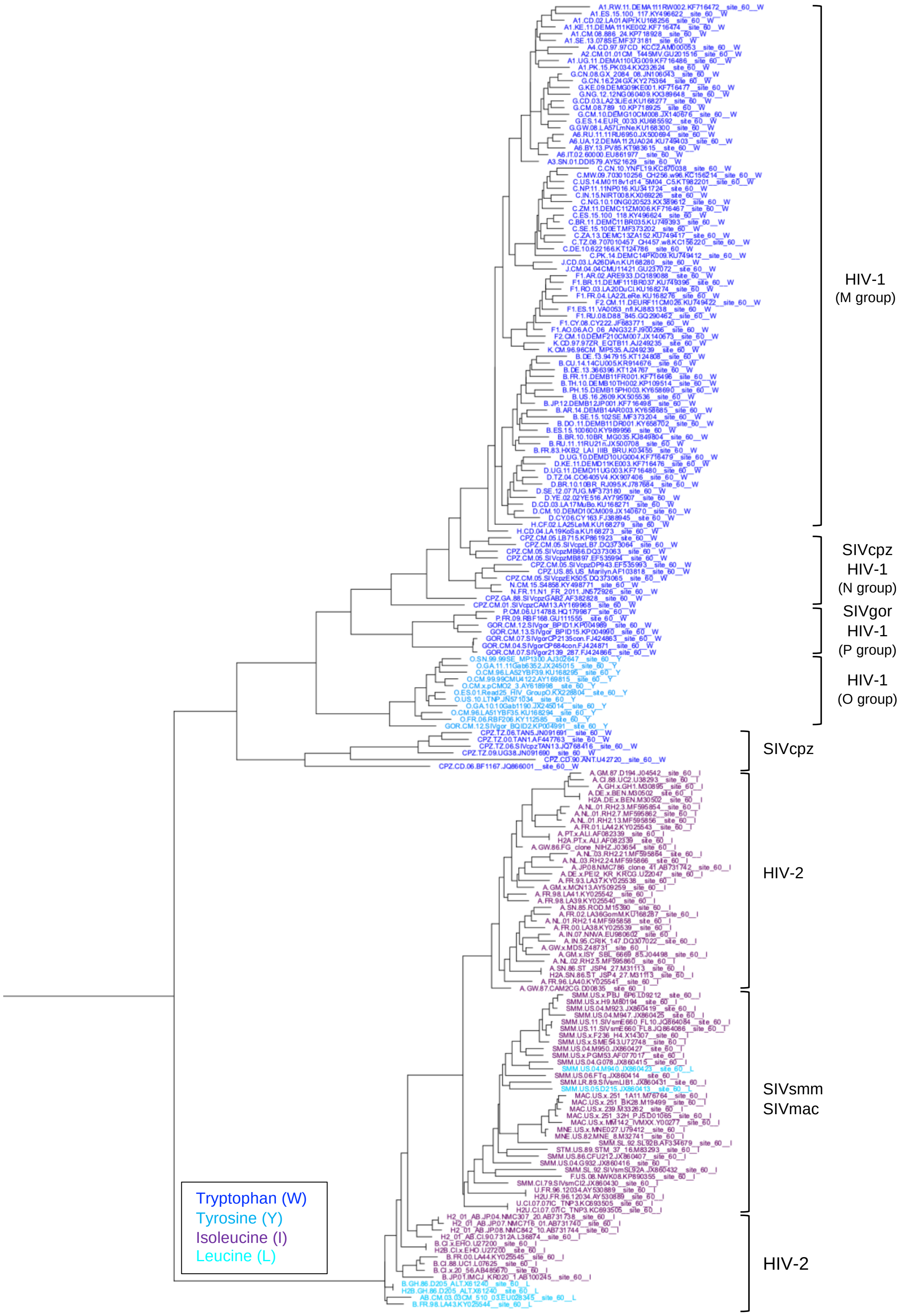

Supplementary Figure S4. Conservation of W757 among HIV and SIV envelope glycoproteins. Phylogenetic analysis of the EnvCT showing amino acid residues found at position 757 (based on 194 consensus envelope sequences from http://www.hiv.lanl.gov/) performed using Chromaclade. Tryptophan (W) – dark blue. Tyrosine (Y) – light blue. Isoleucine (I) – purple. Leucine (L) – turquoise. Related to Figure 7.
